# Cell-integrated serum-induced signalling patterns can differentiate between hand and knee osteoarthritis patients

**DOI:** 10.1093/rheumatology/keae555

**Published:** 2024-11-18

**Authors:** Margot Neefjes, Bas A C Housmans, Charlotte Kaffa, Nathalie G M Thielen, Leo A B Joosten, Cornelia H M van den Ende, Elly L Vitters, Guus G H van den Akker, Tim J M Welting, Arjan P M van Caam, Peter M van der Kraan

**Affiliations:** Department of Rheumatology, Radboud University Medical Center, Nijmegen, The Netherlands; Laboratory for Experimental Orthopedics, Department of Orthopedic Surgery, Maastricht University, Maastricht, The Netherlands; Center for Molecular and Biomolecular Informatics, Radboud University Medical Center, Nijmegen, The Netherlands; Department of Rheumatology, Radboud University Medical Center, Nijmegen, The Netherlands; Department of Internal Medicine, Radboud Institute of Molecular Life Sciences (RIMLS), Radboud University Medical Center, Nijmegen, The Netherlands; Department of Medical Genetics, Iuliu Hatieganu Unversity of Medicine and Pharmacy, Cluj-Napoca, Romania; Department of Rheumatology, Radboud University Medical Center, Nijmegen, The Netherlands; Department of Rheumatology, Sint Maartenskliniek, Nijmegen, The Netherlands; Department of Rheumatology, Radboud University Medical Center, Nijmegen, The Netherlands; Laboratory for Experimental Orthopedics, Department of Orthopedic Surgery, Maastricht University, Maastricht, The Netherlands; Laboratory for Experimental Orthopedics, Department of Orthopedic Surgery, Maastricht University, Maastricht, The Netherlands; Laboratory for Experimental Orthopedics, Department of Orthopedic Surgery, Maastricht University Medical Center, Maastricht, The Netherlands; Department of Rheumatology, Radboud University Medical Center, Nijmegen, The Netherlands; Department of Rheumatology, Radboud University Medical Center, Nijmegen, The Netherlands

**Keywords:** stratification, osteoarthritis, serum, bioassay, endotype

## Abstract

**Objective:**

OA is a very heterogeneous disease. Here, we aimed to differentiate OA patients based on their serum-induced cell-integrated signalling patterns.

**Design:**

In order to monitor the activity of different cellular homeostasis-regulating pathways in response to patient serum, we analysed the response of human OA serum samples to 16 cell-based transcription factor luciferase reporter assays. In this study we compared serum samples from 55 patients with knee OA, 56 patients with hand OA and 42 healthy controls.

**Results:**

Differential serum-induced pathway activity was observed between samples from healthy controls, knee OA and hand OA patients: serum of hand OA patients induced high MAPK-related AP1 activity whereas serum of knee OA patients induced more SRE, ISRE and SOX9 activity, which is related to ELK1-SRF, STAT1-STAT2 and SOX9 activity, respectively. Principal component analysis revealed that these differences differentiate hand OA from knee OA. Both hand and knee OA clustered clearly in two different endotypes each, but no principle component could be identified of these subtypes within either the hand OA or the knee OA sample group.

**Conclusion:**

This study demonstrates that serum from hand OA and knee OA patients evokes diverse cellular signalling patterns that differentiates hand OA, knee OA and healthy controls. This underlines that the pathomolecular mechanisms of OA are likely significantly different between hand and knee OA, a finding that could lead to new insight into the pathobiology of OA endotypes and joint-specific therapies.

Rheumatology key messages

- Sera from hand OA and knee OA patients evoke distinct cellular signalling patterns.
- Transcription factor–based bioassays are suitable for endotyping OA revealing disease subtypes.

## Introduction

OA is the most common joint disease, affecting up to 7% of the global population [1]. Clinically, OA is characterized by pain and loss of joint function, leading to disability. Despite decades of research, there are still no disease-modifying drugs for OA [2]. Besides the problem of our limited knowledge of the regulatory mechanisms of OA development and progression, it has been suggested that the origin of the lack of treatments can be attributed to OA heterogeneity [3, 4]. OA has a multifactorial nature, with risk factors including age, sex, previous joint injury and obesity [5]. Besides this, OA can also affect different anatomical joints. Therefore, there is a high clinical need for effective methods that can stratify OA in different subtypes and identify underlying pathological processes. Identification and characterization of these OA subtypes is essential for the development of personalized treatment strategies.

In recent years, studies have attempted to stratify OA patients based on clinical phenotypes, such as inflammatory, metabolic and mechanical OA [6, 7]. However, these subgroupings have not yet led to breakthroughs in treatment. A possibly more promising way to stratify OA is by the use of endotypes. An endotype is defined as a subtype of disease where the pathophysiology can be explained by the involvement of certain (distinct) molecular pathways [4]. Several studies have stratified OA in a number of endotypes, based on the transcriptomic profiles of different joint tissue biopsies (e.g. cartilage, synovium and bone) [8–10]. This stratification is very valuable as it offers insight into to underlying disease mechanisms and can provide clues for therapeutic targets in a specific patient population. However, clinical implementation of such an endotyping approach is challenging due to the invasiveness of a biopsy and the extra damage it can cause. Therefore, a less invasive method would be preferred, ideally a blood-based assay, e.g. using serum. Serum is a blood component, which can be easily collected and therefore is an ideal candidate for the use in diagnostic purposes.

To tackle the heterogeneous aspect of OA, we questioned whether serum from hand OA, knee OA or healthy controls (HCs) induced distinct cell-integrated signalling patterns indicative of endotypes. For this purpose, we generated a screening platform consisting of 16 cell-based transcription factor luciferase reporter assays, which allowed us to monitor various cellular homeostasis-regulating pathways in response to patient serum.

## Methods

### Human serum collection

Serum was collected from 55 knee OA patients and 56 hand OA patients as part of randomized, double-blinded, sham-controlled superiority trial to study the effects of low-dose radiation therapy [11, 12]. We analysed serum that was collected at baseline (T0, before start intervention). Baseline characteristics of OA patients were similar between the two groups (Table 1). The local Medical Ethics committee approved the study (2014–275). All patients gave written informed consent. Serum from 42 healthy subjects of the 200 FG cohort (www.humanfuntionalgenomics.org) was used in this study [13]. The ethnicity distribution of both OA cohorts and the healthy subjects was all white.

**Table 1.** Patient demographics of serum donors

Clinical parameters	Hand OA	Knee OA	HC I
*N*	56	55	42
Age (years), mean (s.d.)	65 (6.6)	65 (9)	61 (7)
Women, *n* (%)	44 (79)^a^	28 (51)	15 (36)
BMI (kg/m^2^), mean (s.d.)	28 (4.6)	28 (5)	
Kellgren and Lawrence ≥2, *n* (%)	27 (48)	32 (58)	
Duration of symptoms, *n*			
1–3 years	10	17	
3–5 years	12	13	
5–10 years	13	11	
10–15 years	8	6	
>15 years	13	8	
NRS pain score (0–10), mean (s.d.)	6 (1.7)	6 (2)	
VAS pain	6 (2)	6 (2)	
AUSCAN pain (0–100)	55 (16.9)		
WOMAC pain (0–100)		60 (15)	
AUSCAN function (0–100)	57 (21)		
WOMAC function (0–100)		61 (18)	
ESR (mm/h)	14 (9.5)	13 (8)	
CRP (mg/l)	4 (9.4)	3 (6)	
Smoking, *n*			
Yes	7^b^	3	
No	49	52	

a The hand OA group contains significantly more women than the knee OA and HC groups.

b Not significantly higher than knee OA group. NRS: numerical rating scale; VAS: visual analogue scale; AUSCAN: Australian/Canadian OA hand index

### Construction reporter plasmids and virus production

Binding sequences (Table 2) specific for 14 transcription factors were extended with a minimal promoter and were synthesized by Genecust (Boynes, France). Binding sequences were directionally cloned into the pNL1.2 vector (Promega, Madison, WI, USA). Subsequently, lentiviral constructs were generated by re-cloning pNL1.2 reporters with the In-Fusing Cloning method (TakaraBio, Kusatsu, Shiga, Japan) into the ClaI site of the pLVX-EF1α-IRES-Puro producer vector (TakaraBio). The 4th generation lentiviral production system (TakaraBio) was used to generate viral supernatants in Lenti-X HEK 293 T cells (TakaraBio). Polyethylenimine (PEI; Polysciences, Warrington, USA) 1 mg/ml was used to transfect viral constructs into Lenti-X 293 T cells. Viral supernatant collected after 48 and 72 h post-transfection were concentrated using ultracentrifugation (2 h, 25 000 × *g*, 4°C). Lentiviral titre was determined by p24 ELISA (Fujirebio, Gent, Belgium).

**Table 2.** Transcription factor reporter element sequences

Transcription factor element	Sequence
AP1, Activator protein 1 response element	TGAGTCAGTGACTCAGTGAGTCAGTGACTCAGTGAGTCAGTGACTCAGCTCGAGGATATCAAGATCTGGCCTCGGCGGCCAAGCTTAGACACTAGAGGGTATATAATGGAAGCTCGACTTCCAG
ARE, Antioxidant response element	TAGCTTGGAAATGACATTGCTAATGGTGACAAAGCAACTTTTAGCTTGGAAATGACATTGCTAATGGTGACAAAGCAACTTTCTCGAGGATATCAAGATCTGGCCTCGGCGGCCAAGCTTAGACACTAGAGGGTATATAATGGAAGCTCGACTTCCAG
CRE, Cyclic AMP response element	TGACGTCAGCTGCCAGATCCCATGGCCGTCATACTGTGACGTCTTTCAGACACCCCATTGACGTCAATGGGAGAACAGATCTGGCCTCGGCGGCCAAGCTTAGACACTAGAGGGTATATAATGGAAGCTCGACTTCCAG
GRE, Glucocorticoid receptor element	AGAACATTTTGTCCGAGAACATTTTGTCCGAGAACATTTTGTCCGAGAACATTTTGTCCGAGAACATTTTGTCCGAGAACATTTTGTCCGCTCGAGGATATCAAGATCTGGCCTCGGCGGCCAAGCTTAGACACTAGAGGGTATATAATGGAAGCTCGACTTCCAG
ISRE, Interferon-stimulated response element	TAGTTTCACTTTCCCTAGTTTCACTTTCCCTAGTTTCACTTTCCCTAGTTTCACTTTCCCTATTTCACTTTCCCCTCGAGGATATCAAGATCTGGCCTCGGCGGCCAAGCTTAGACACTAGAGGGTATATAATGGAAGCTCGACTTCCAG
NFAT5, Nuclear factor of activated T cells 5 response element	TGGAAAAGTCCATGGAAAAGTCCATGGAAAAGTCCATGGAAAAGTCCATGGAAAAGTCCATGGAAAAGTCCATGGAAAAGTCCATGGAAAAGTCCACTCGAGGATATCAAGATCTGGCCTCGGCGGCCAAGCTTAGACACTAGAGGGTATATAATGGAAGCTCGACTTCCAG
NFκB, Nuclear factor k B response element	GGGAATTTCCGGGGACTTTCCGGGAATTTCCGGGGACTTTCCGGGAATTTCCAGATCTGGCCTCGGCGGCCTAGATGAGACACTAGAGGGTATATAATGGAAGCTCGACTTCCAG
PPRE, Peroxisome proliferator activated receptor-y response element	GTCGACAGGGGACCAGGACAAAGGTCACGTTCGGGAGTCGACAGGGGACCAGGACAAAGGTCACGTTCGGGAGTCGACAGGGGACCAGGACAAAGGTCACGTTCGGGAGTCGACCTCGAGGATATCAAGATCTGGCCTCGGCGGCCAAGCTTAGACACTAGAGGGTATATAATGGAAGCTCGACTTCCAG
SBE, SMAD binding element	AGTATGTCTAGACTGAAGTATGTCTAGACTGAAGTATGTCTAGACTGACTCGAGGATATCAAGATCTGGCCTCGGCGGCCTAGATGAGACACTAGAGGGTATATAATGGAAGCTCGACTTCCAG
SIE, Sis inducible element	AGCTTCATTTCCCGTAAATCGTCGAAGCTTCATTTCCCGTAAATCGTCGAAGCTTCATTTCCCGTAAATCGTCGAAGCTTCATTTCCCGTAAATCGTCGAAGCTTCATTTCCCGTAAATCGTCGACTCGAGGATATCAAGATCTGGCCTCGGCGGCCAAGCTTAGACACTAGAGGGTATATAATGGAAGCTCGACTTCCAG
SOX9, SRY-box transcription factor 9 response element	AGAACAATGGAGAACAATGGAGAACAATGGAGAACAATGGAGAACAATGGAGAACAATGGAGAACAATGGCTCGAGGATATCAAGATCTGGCCTCGGCGGCCAAGCTTAGACACTAGAGGGTATATAATGGAAGCTCGACTTCCAG
SRE, Serum response element	AGGATGTCCATATTAGGACATCTAGGATGTCCATATTAGGACATCTAGGATGTCCATATTAGGACATCTAGGATGTCCATATTAGGACATCTAGGATGTCCATATTAGGACATCTAGATCTGGCCTCGGCGGCCAAGCTTAGACACTAGAGGGTATATAATGGAAGCTCGACTTCCAG
SRF, Serum response factor	AGTATGTCCATATTAGGACATCTACCATGTCCATATTAGGACATCTACTATGTCCATATTAGGACATCTTGTATGTCCATATTAGGACATCTAAAATGTCCATATTAGGACATCTAGATCTGGCCTCGGCGGCCAAGCTTAGACACTAGAGGGTATATAATGGAAGCTCGACTTCCAG
TCF/LEF, T cell factor/lymphoid enhancer factor family response element	AGATCAAAGGGTTTAAGATCAAAGGGCTTAAGATCAAAGGGTATAAGATCAAAGGGCCTAAGATCAAAGGGACTAAGATCAAAGGGTTTAAGATCAAAGGGCTTAAGATCAAAGGGCCTACTCGAGGATATCAAGATCTGGCCTCGGCGGCCAAGCTTAGACACTAGAGGGTATATAATGGAAGCTCGACTTCCAG

Underlined is the minimal promoter including a TATA-box, which is the same for every sequence.

### SW1353 cell culture and stable cell-line generation

SW1353 cells (ATCC, HTB-94) were cultured in growth medium consisting of Dulbecco’s Modified Eagle Medium/Nutrient Mixture F-12 (DMEM/F12; ThermoFisher, Carlsbad, CA, USA) supplemented with 10% fetal calf serum (FCS; Sigma, St Louis, Missouri, USA) and 1% penicillin-streptomycin-glutamine (Gibco, Carlsbad, CA) in a humidified atmosphere containing 5% CO_2_ at 37°C. SW1353 reporter cell lines were generated using lentiviral transduction (250 ng virus particles/62 500 cells) with 8 µg/ml hexadimethrine bromide (Sigma) for 8 h. Two days post-transduction, cells were selected using 1 µg/ml puromycin (Sigma) and expanded for three passages before cryopreservation and stored in liquid nitrogen until further use. All 14 reporter cell lines were functionally validated with known positive stimuli (Supplementary Fig. S1, available at *Rheumatology* online).

### Stimulation and transcription factor luciferase reporter assay

SW1353 reporter cell lines were defrosted and expanded for another two passages before the start of the experiment. Cells were trypsinized and re-seeded (95 250 cells/cm^2^) into white polystyrene 384-well plates (Greiner Bio-One, Alpen aan den Rijn, The Netherlands) and serum-starved overnight in 0% FCS supplemented DMEM/F12. Serum-starved cells were stimulated with 10% OA serum (*n* = 111 donors) or HC serum (*n* = 42 donors) for 6 h. After stimulation cells were lysed using 15 µl ultrapure H_2_O. Nano-Glo (Promega) was added at 1:1 ratio to the cell lysate to measure luminescence. All luminescent measurements were performed with the CLARIOstar (BMG Labtech) at room temperature.

### Data analysis

Luciferase reporter measurement were investigated in quadruple. Fold-change (FC) data from reporter measurements were calculated by consecutively subtracting the background signal and normalizing to the mean of the unstimulated negative control conditions. To test whether luciferase FC was different between groups, a (grouped) mixed effect model was used and to control the false-discovery rate we used the two-stage linear step-up method of Benjamini, Krieger and Yekutieli with a Q of 0.05 (GraphPad Prism 9.3.1). The generated p and q values are listed in Supplementary Table S1, available at *Rheumatology* online. Data were considered statistically significant when *P* ≤ 0.05. Each dot represents mean of quadruple measurement.

Heatmaps and principal component analysis (PCA) plots were created using R (v. 4.1.2), using packages readxl (v. 1.3.1), pheatmap (v. 1.0.12), ggfortify (v. 0.4.14) and PCAtools (v. 2.6.0). The reporter FCs were read into R with the read_xlsx function. In figures where data were transformed a log2 transformation was applied.

## Results

### Serum-induced pathway activity is different between samples from healthy, knee OA and hand OA donors

**Figure 1.**
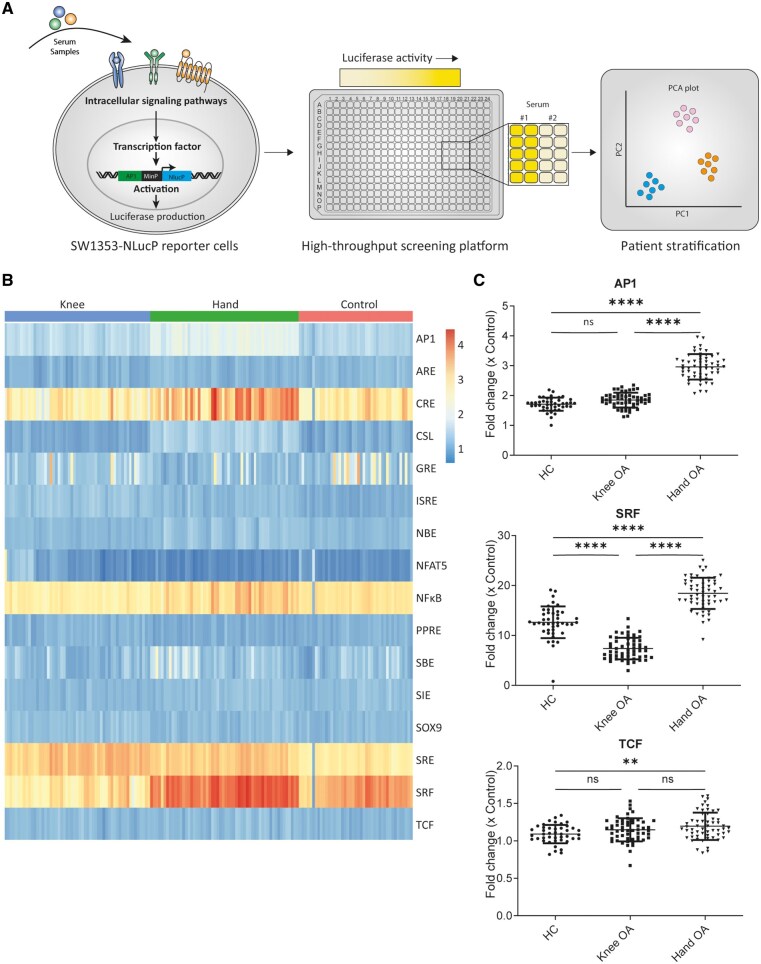
Serum-induced pathway activity different between samples from healthy controls, knee OA and hand OA donors. (**A**) Schematic depiction of experimental design. (**B**) Log-transformed clustered heatmap of response of all 153 serum samples (*n* = 55 knee OA patients, *n* = 56 hand OA patients, *n* = 42 healthy control donors) on transcription factor response element reporters. (**C**) Comparison of AP1, SRF and TCF reporter response between healthy control, knee OA and hand OA samples. Fold-change data from reporter measurements were calculated by consecutively subtracting the background signal and normalizing to the mean of the unstimulated negative control conditions. To test whether luciferase fold change was different between groups, one-way analysis of variance was used with Tukey’s multiple comparison test. Each dot represents mean of quadruple measurement. ns: not significant; *P-value ≤0.01; **P-value ≤0.0001

To investigate the heterogeneous aspect of OA, we investigated whether serum from knee OA, hand OA or HCs induced distinct cell-integrated signalling patterns. Sixteen SW1353 reporter cell lines were validated with known positive stimuli (Supplementary Fig. S1, available at *Rheumatology* online). Fourteen transcription factor reporters were significantly upregulated by known positive stimuli, while two reporters (CSL and NBE) were not. To identify potential serum-induced differences between hand OA, knee OA and HCs, we performed functional pathway analysis of 153 serum samples (hand *n* = 55, knee *n* = 56, HC *n* = 42) using our transcription factor response element reporter screening platform (Fig. 1A).

A clustered heatmap of log-transformed luciferase reporter data revealed differences in specific reporter outcome between hand OA, knee OA and HC (Fig. 1B). Overall CRE, NFκB, SRE and SRF reporters demonstrated the highest inducibility by all different groups, while ARE, CSL, NBE, NFAT5, PPRE, SIE, SOX9 and TCF displayed only minor FCs by any of the groups (FC ≤2.0). However, for all reporters, significant differences were found between at least two of the groups (Fig. 1C; Supplementary Fig. S2, available at *Rheumatology* online). For example, the hand OA serum-induced response of the AP1 reporter (FC 3.0 ± SD 0.4) was significantly higher than the induced response by HC serum (FC 1.7 ± SD 0.2) and knee OA. However, knee OA–induced response of the AP1 reporter (FC 1.8 ± SD 0.2) did not significantly differ from HC serum. For the SRF reporter, knee OA serum induced a significantly lower response (FC 7.2 ± 2.2) compared with HC serum (FC 12.6 ± 3.2) and hand OA serum (FC 18.4 ± 3.1). Lastly, for the TCF reporter, only serum from hand OA (FC 1.2 ± 0.2) induced a significantly higher response than HC serum (FC 1.1 ± 0.1), but no difference was observed between knee OA serum and HC and knee OA serum and hand OA serum. Furthermore, magnitude of induced serum responses differed greatly between transcription factor response element reporters (Fig. 1C; Supplementary Fig. S2, available at *Rheumatology* online). For example, AP1 showed an average inducibility (highest FC 4.0), while SRF reporter showed the highest inducibility (highest FC 25.1) and TCF showed a very weak inducibility (highest FC 1.6). In total, 14 out of 16 reporters (TCF and GRE not) revealed a significant difference between stimulation with hand OA and knee OA serum, indicating that serum is indeed different between OA patients.

### Serum-induced signalling patterns can stratify hand OA, knee OA and HCs

**Figure 2.**
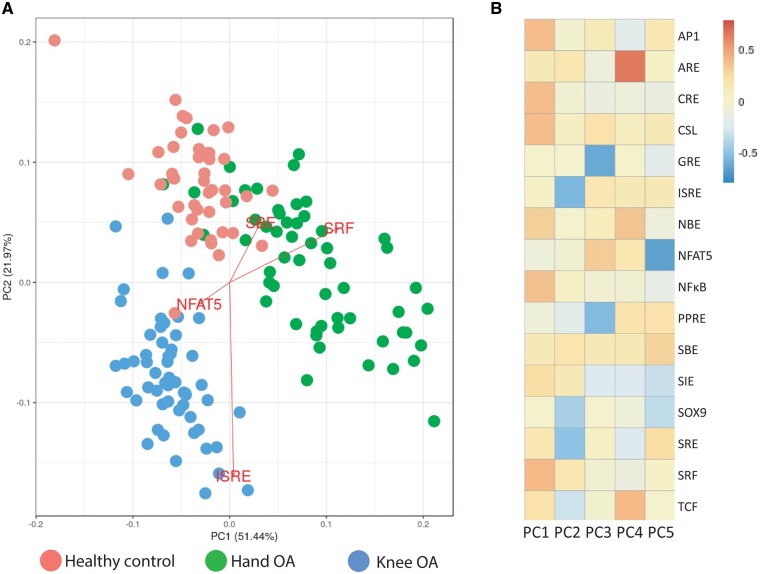
Serum-induced signalling patterns can stratify hand OA, knee OA and healthy controls. (**A**) Principal component analysis (PCA) plot of all 153 serum samples. (**B**) Visualization of the transcription factors reporters that explain the variance of the principal components 1–5 (PC1–PC5)

To investigate if these observed differences between serum-induced signalling patterns could be used to differentiate phenotypes, we performed a PCA. The first two components of PCA (which explained 73.4% of total variance) clearly separated two cohorts (Fig. 2A). Knee OA clustered separately from HCs and hand OA, primarily by ISRE and NFAT reporter activity. In addition, hand OA patients and HCs showed a small overlap in the PCA plot due to large variability in the hand OA group. Still, individual clouds could be determined and these were mainly separated by SRF and SRE. Next, we investigated whether the variance explained by a principal component (PC) is the result of one or more reporters (Fig. 2B). PC1, explaining 51.4% of the variance, is mainly determined by six different reporters (AP1, CRE, CSL, NBE, NFκB and SRF). PC2, explaining 21.9% of the variance, is predominantly determined by four reporters (ISRE, SOX9, SRE and TCF).

### No subtypes can be identified by the serum-induced signalling patterns within the hand OA and knee OA group

**Figure 3.**
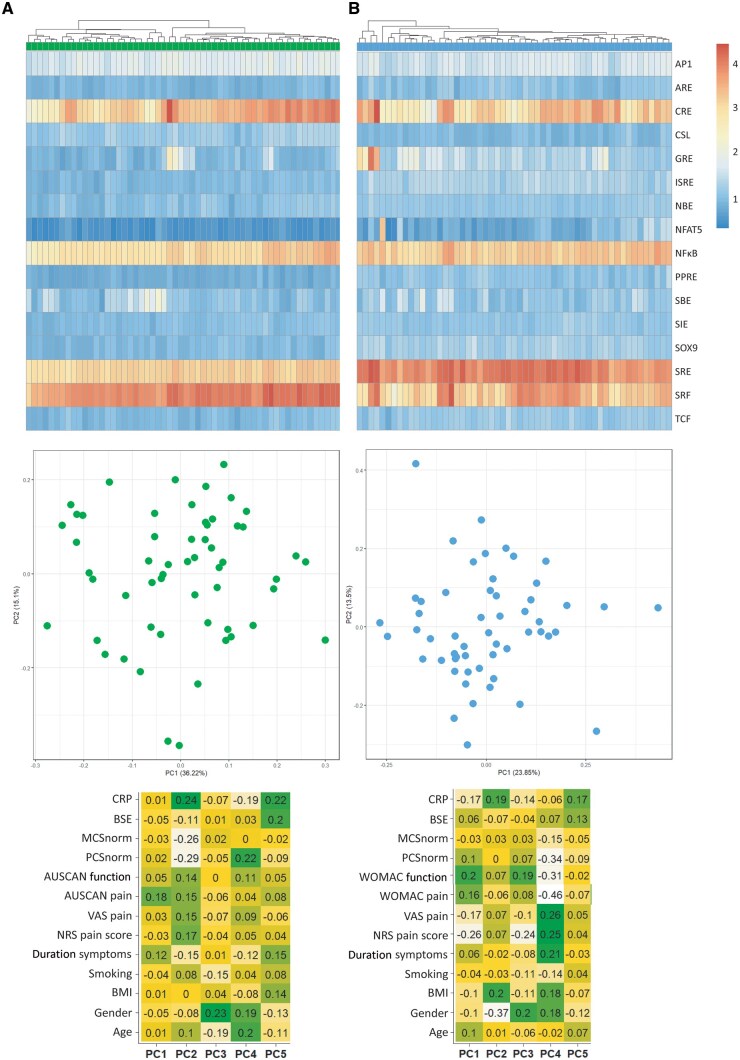
No subtypes can be identified by serum-induced signalling patterns within either the hand OA or knee OA group. (**A**) Log-transformed clustered heatmap of response of hand OA patients on transcription factor response element reporters. Principal component analysis (PCA) plot of hand OA samples and correlation of principal components to different clinical parameters. (**B**) Log-transformed clustered heatmap of response of knee OA patients on transcription factor response element reporters. PCA plot of hand OA samples and correlation of principal components to different clinical parameters

Next, we questioned whether we could also identify joint specific subtypes. Therefore, we generated a clustered heatmap of log-transformed luciferase reporter data (Fig. 3A and B). The dendrogram split the hand OA patients into two separate groups (*n* = 25 and *n* = 21 per group; Fig. 3A). However, in the PCA plot no clear separate groups are visible. Next, we investigated if the variation within the hand OA samples could be explained by a more simplistic clinical parameter. Hence, we correlated the PCs to a list of clinical parameters (e.g. age, gender, NRS pain, etc.). No significant correlation could be identified between the PCs and a clinical parameter. Likewise, for knee OA the dendrogram split the patient group into two (*n* = 4 and *n* = 51 per group; Fig. 3B). Additionally, no separate groups were visible in the PCA plot and no significant correlation were found between PCs and clinical parameters.

## Discussion

OA is a very heterogeneous disease, with most likely different underlying molecular mechanisms that drive the disease. This is thought to be one of the main reasons why there is no disease-modifying drug available yet. Here, we aimed to clarify the heterogeneous aspect of OA by investigating whether the cell-integrated signalling patterns induced by serum from either hand OA patients, knee OA patients or HCs could help stratify them into groups and identify underlying disease processes (endotypes). We report that serum-induced cellular signalling pathway activity is different between samples from healthy, knee OA and hand OA donors and that this signalling pattern can be used to differentiate these patients. Furthermore, we investigated whether this method could be used to stratify patients within one joint group into different subtypes, but we could not confirm this in our groups with the current relatively small sample size.

In recent years there have been multiple studies investigating the difference between OA in different joints. There have been multiple animal studies in which evidence suggests that the molecular pathophysiology is different between joints, especially knee and hip OA [14–16]. In addition, studies with human samples revealed that the serum and SF cytokine profile is different between hip and knee OA [17]. Furthermore, the inflammatory processes in the synovium are also different between hip and knee OA as is the mononuclear cell population and their cytokine release profiles [18]. For example, synovial membrane cells isolated from hip tissue biopsies released higher concentrations of inflammatory cytokines, such as IL-17, TNF-α and IFN-γ, than those from knee biopsies. Besides differences between hip and knee OA, differences have also been reported between knee and post-traumatic wrist OA. Teunis *et al.* [19] described general higher inflammatory levels (e.g. IL-6 and IL-17) in SF from posttraumatic wrist OA compared with SF from primary knee OA. Furthermore, SF from knee OA and hand OA differed in protein composition and the SF provoked significant different responses in cytokine release, cell death and hypertrophy [20].

In the previously discussed studies, mainly SF was used. A disadvantage of SF is that it is not as easily accessible as serum. However, multiple efforts have been made to find a biomarker for patient stratification in serum, but none has proven successful for in-clinic use [21–24]. Most of these studies have focused on a single molecule as biomarker, but we think this approach very challenging in the case of OA as it is an extremely complex disease. We propose that a solution might be to investigate a panel of, e.g. cytokines or activated cell signalling pathways. Furthermore, the advantage of our method, a bioassay, is that it is a cell-based system in which the cell itself integrates all the signals that are presented by the stimulus, which mimicks the *in vivo* situation.

We demonstrated that based on 16 cell-based transcription factor luciferase reporter assays, we could subgroup serum derived from hand OA patients, knee OA patients and HCs. Furthermore, we have shown that the PCs are explained not by a single reporter assay, but by the combination of multiple reporters. This highlights again both the complexity of the disease as well as the need for more advanced techniques to identify signalling patterns/expression patterns (signatures) that can help us to better understand the molecular mechanisms driving OA. For example, our data point towards the use of a combination of the AP1, CRE, CSL, NBE, NFκB and SRF reporters as these together explained 51.4% of the variance of PC1. Of note, in this study we used the SW1353 chondrosarcoma cell line. We chose for this cell type because we have validated that positive controls induce activity of the designed transcription factor luciferase constructs in this cell type. This is important because each transcription factor is not present or active in every cell type, and results of transcription factor luciferase reporter assays can differ between cell types. However, a drawback of this cell line is that it is not a chondrocyte, making interpretation of our results in context of chondrocyte biology more difficult.

A limitation of this study is that serum samples from only one patient cohort were investigated, and that we could only match these cohorts for age, and not for sex, BMI or smoking. Furthermore, we cannot fully exclude that some patients had both hand and knee OA, because patients were not clinically evaluated for (subclinical) OA in other joints than the one they were included for. These factors could be confounding the results. Therefore, the subgroups defined herein need to be validated by a second, independent cohort of patients in which there is better matching of the patient and control groups. Only then can the true discriminative capacity of our bioassay be confirmed. Furthermore, serum samples used in this study were derived from a clearly defined patient group (e.g. age ≥50 years and pain score ≥5/10) and it would be highly interesting to investigate whether our developed bioassay also has potential to identify early OA and other types of OA such as post-traumatic OA. Serum also comes with the limitation that the differences we observe are not necessarily joint-derived, but can also be the result of a systemic response of other organs to the disease process or be confounded by the physiological condition of the patient due to, e.g. fasting, recent exercise or pain killer use. This makes interpretation of these findings in context of the pathophysiology of OA more difficult. Another limitation of our study is that the ethnicity of both the OA and healthy volunteer cohorts was all white, which possibly limits the value of our observations in people of other ethnicities.

We could not identify different subgroups within either the knee or the hand OA group. We found indications that differences exist, however we could not clearly uncover distinct groups. This may be attributed to the relatively low number of samples per group (hand OA *n* = 55, knee OA *n* = 56). Further investigations are needed, with larger cohorts, to determine whether our developed reporter assay can also be used for joint-specific patient stratification.

In conclusion, our novel OA serum induced cell-integrated signalling screening method was able to differentiate knee OA, hand OA and HC serum. These distinct OA types may require different pharmacological interventions [25]. For this, it will be interesting to further investigate intracellular pathways and upstream effectors that drive the activation of the identified transcription factors. In addition, further refinement of the current cell-based OA endotyping tool could improve personalized medicine approaches.

## Supplementary Material

keae555_Supplementary_Data

## Data Availability

The Radboudumc endorses FAIR data management. All data are available upon reasonable request.
