# Supplementary material for: Cell-integrated serum-induced signalling patterns can differentiate between hand and knee osteoarthritis patients
Source: Rheumatology (Oxford). 2024 Nov 18;64(6):3929–37. doi: 10.1093/rheumatology/keae555 (PMC12107062; doi:10.1093/rheumatology/keae555)
Supplement: keae555_Supplementary_Data [file keae555_supplementary_data.zip › keae555_Supplementary_Data/rhe-23-2136-File007.docx]

**Supplementary figure 1**

**
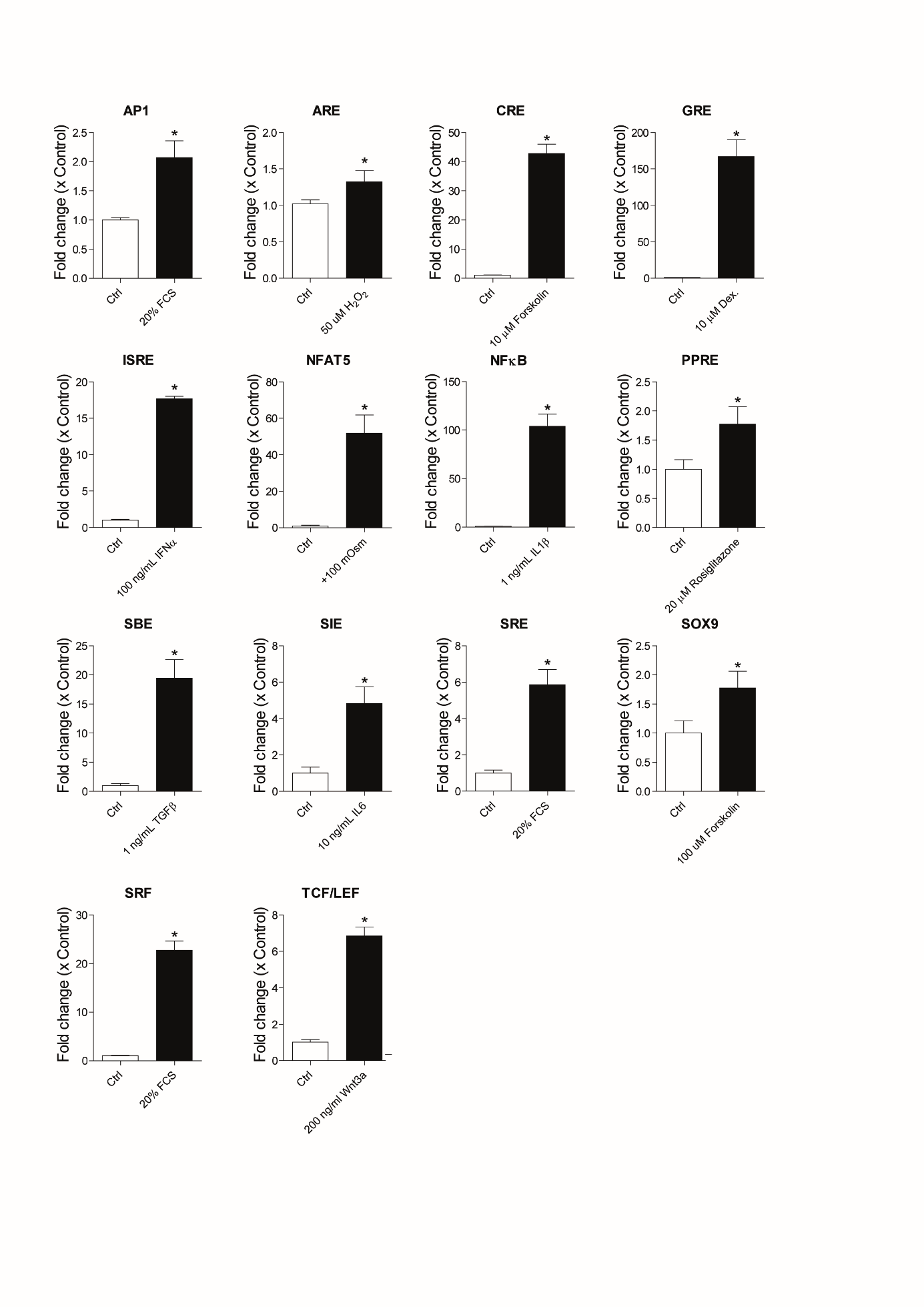
**

**Supplementary Figure 1. Transcription factor reporter cell lines validated with positive stimuli.** The TF reporter cell lines (SW1353) were tested for responsiveness by known positive stimuli. * p. value ≤0.05. FCS, fetal calf serum; Dex, dexamethasone; IFNα, interferon alpha; IL1β, interleukin 1 beta; TGFβ1, transforming growth factor beta; IL6, interleukin 6; +Osm increase in NaCl.

**Supplementary figure 2**

**
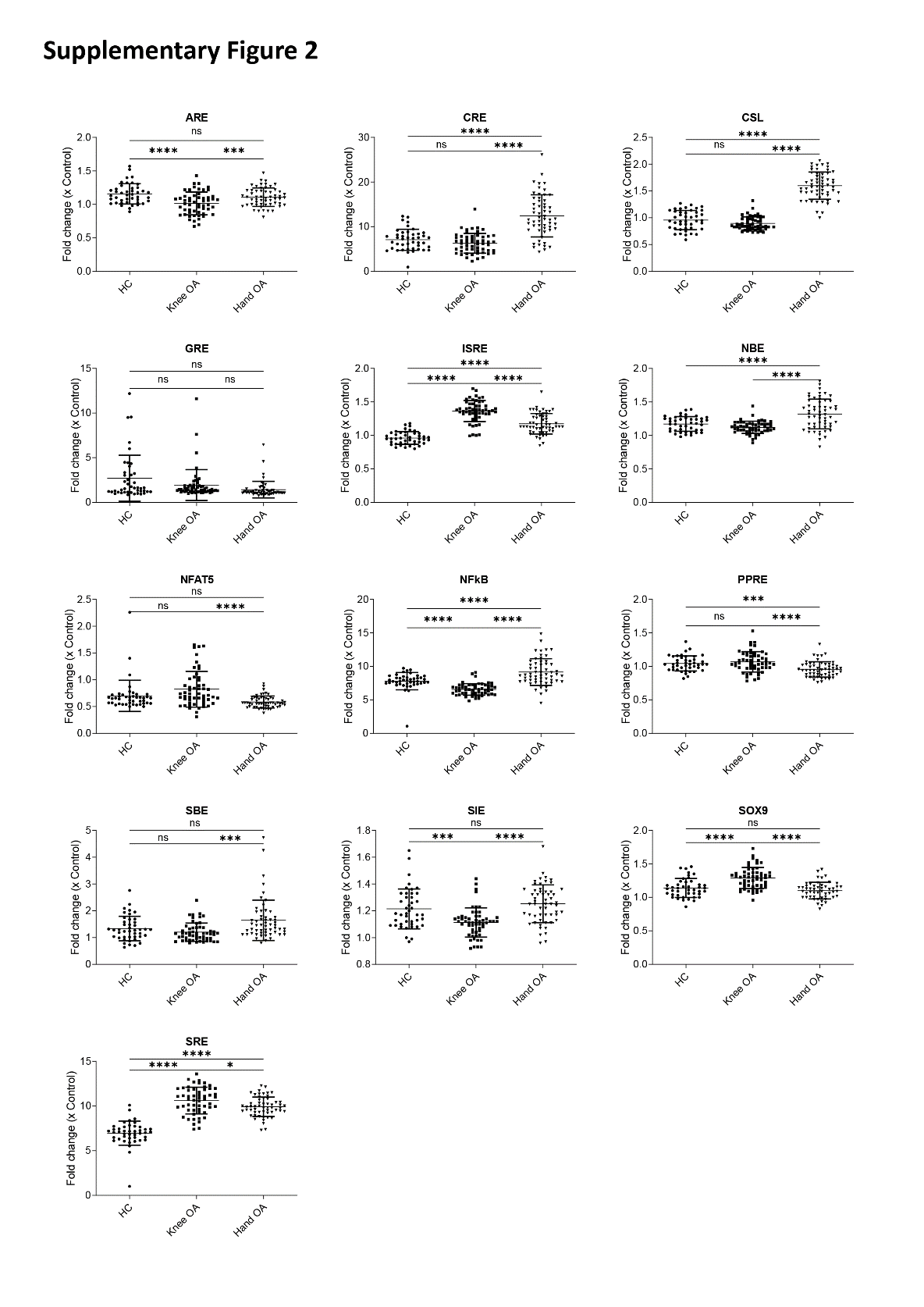
**

**Supplementary Figure 2. Serum-induced pathway activity different between samples from healthy controls, knee OA and hand OA donors.** Comparison of ARE, CRE, CSL, GRE, ISRE, NBE, NFAT5, NFκB, PPRE, SBE, SIE, SOX9 and SRE reporter response between healthy control, knee OA and hand OA samples. Fold change data from reporter measurements were calculated by consecutively subtracting the background signal and normalizing to the mean of the unstimulated negative control conditions. To test if luciferase fold change was different between groups, we used a (grouped) mixed effect model and to control the FDR we used the Two-stage linear step-up method of Benjamini, Krieger and Yekutieli with a Q of 0.05 (GraphPad Prism 9.3.1). The generated p and q values are now **listed in supplementary data table 1.** Each dot represents mean of quadruple measurement. ns= not significant, * p. value ≤0.05, ** p. value ≤0.01, *** p. value ≤0.001 and **** p. value ≤0.0001.

Supplementary data table 1; p and q values of serum screening in Suppl figure 2
